# Cell Surface Expression of Endosomal Toll-Like Receptors—A Necessity or a Superfluous Duplication?

**DOI:** 10.3389/fimmu.2020.620972

**Published:** 2021-02-01

**Authors:** Matylda Barbara Mielcarska, Magdalena Bossowska-Nowicka, Felix Ngosa Toka

**Affiliations:** ^1^ Division of Immunology, Institute of Veterinary Medicine, Department of Preclinical Sciences, Warsaw University of Life Sciences, Warsaw, Poland; ^2^ Center for Integrative Mammalian Research, Department of Biomedical Sciences, Ross University School of Veterinary Medicine, Basseterre, Saint Kitts and Nevis

**Keywords:** TLR3, cell surface expression, nucleic acid-sensing receptors, TLR signaling, innate immune function

## Abstract

Timely and precise delivery of the endosomal Toll-like receptors (TLRs) to the ligand recognition site is a critical event in mounting an effective antimicrobial immune response, however, the same TLRs should maintain the delicate balance of avoiding recognition of self-nucleic acids. Such sensing is widely known to start from endosomal compartments, but recently enough evidence has accumulated supporting the idea that TLR-mediated signaling pathways originating in the cell membrane may be engaged in various cells due to differential expression and distribution of the endosomal TLRs. Therefore, the presence of endosomal TLRs on the cell surface could benefit the host responses in certain cell types and/or organs. Although not fully understood why, TLR3, TLR7, and TLR9 may occur both in the cell membrane and intracellularly, and it seems that activation of the immune response can be initiated concurrently from these two sites in the cell. Furthermore, various forms of endosomal TLRs may be transported to the cell membrane, indicating that this may be a normal process orchestrated by cysteine proteases—cathepsins. Among the endosomal TLRs, TLR3 belongs to the evolutionary distinct group and engages a different protein adapter in the signaling cascade. The differently glycosylated forms of TLR3 are transported by UNC93B1 to the cell membrane, unlike TLR7, TLR8, and TLR9. The aim of this review is to reconcile various views on the cell surface positioning of endosomal TLRs and add perspective to the implication of such receptor localization on their function, with special attention to TLR3. Cell membrane-localized TLR3, TLR7, and TLR9 may contribute to endosomal TLR-mediated inflammatory signaling pathways. Dissecting this signaling axis may serve to better understand mechanisms influencing endosomal TLR-mediated inflammation, thus determine whether it is a necessity for immune response or simply a circumstantial superfluous duplication, with other consequences on immune response.

## Introduction

TLR3, like all members of the Toll-like receptor family, recognizes pathogen-associated molecular patterns (PAMPs), danger-associated molecular patterns (DAMPs), and plays an essential role in innate immunity. While origins of microbial derivatives of TLR ligands are straightforward, endosomal TLRs can recognize self-nucleic acids emerging, e.g., during tissue damage caused by UV-radiation or from non-apoptotic cell debris (1). The importance of TLR3 in self-RNA recognition was discussed in the work of Takemura et al. (2). where high-dose ionizing radiation severely affected murine epithelial stem cells of small intestine, causing the gastrointestinal syndrome (GIS) Damage of nucleic acids and leakage of cellular RNA from the cells activated TLR3 which proved to be critical to the pathogenesis of the disease as *Tlr3^-/-^* mice showed significant resistance to GIS. Nevertheless, host-derived TLR ligands may be present in the extracellular environment as well as in endosomes, however, they undergo rapid degradation by nucleases, reducing the risk of autoimmune or autoinflammatory disorders (3). Although mechanisms that control the precise transportation of the endosomal TLRs to the ligand recognition site are strictly regulated, barriers can be overcome and lead to autoimmune diseases such as lupus erythematosus (4), psoriasis (5), or rheumatoid arthritis (6).

TLRs may be classified according to their cellular localization, as they may occur on the cell surface or in the membranes of intracellular vesicles referred to as endosomes. All endosomal TLRs identified in mice and humans: TLR3, TLR7, TLR8, and TLR9, sense nucleic acids or their derivatives, i.e., double-stranded RNA (dsRNA), single-stranded-RNA (ssRNA), uridine-rich or uridine/guanosine-rich ssRNA, and unmethylated CpG DNA respectively (7, 8). The size of human endosomal TLRs is about 1000 amino residues, compared to cell surface-localized TLRs which have approximately 800 amino acids [see **Figure 2** in (5)]. Although TLRs are acknowledged as evolutionarily highly conserved proteins, current studies indicate that TLR3 is the most conserved innate receptor compared to TLR7, TLR8, and TLR9 (9, 10).

Endosomal TLRs are subjected to many elaborate regulations, especially related to transportation and localization in the cell. Recent findings dispute the dogma that TLR3, TLR7, TLR8, and TLR9 are exclusive intracellular receptors. Although the endosomal acidic environment is crucial for the functioning of endosomal TLRs (11, 12), surprisingly, the same receptors may appear on the surface of various cell types and they may trigger signaling pathways (13–16). However, mechanisms leading to and managing such transposition remain obscure. In this review, we sought to reconcile scientific evidence indicating specific conditions that support membrane positioning of endosomal TLRs, particularly TLR3, and outline factors contributing to TLR3 occurrence in the plasma membrane. Insights into TLR biology regarding receptor transportation may permit full comprehension of the impact of receptor localization on its function. Furthermore, highlighting similarities and differences between various cell types may yield valuable knowledge on individual TLRs, regarding therapeutic targets for diseases that may result from receptor localization abnormalities.

## Structure of Endosomal TLRs and Effect on the Localization in the Cell

**Figure 1:**
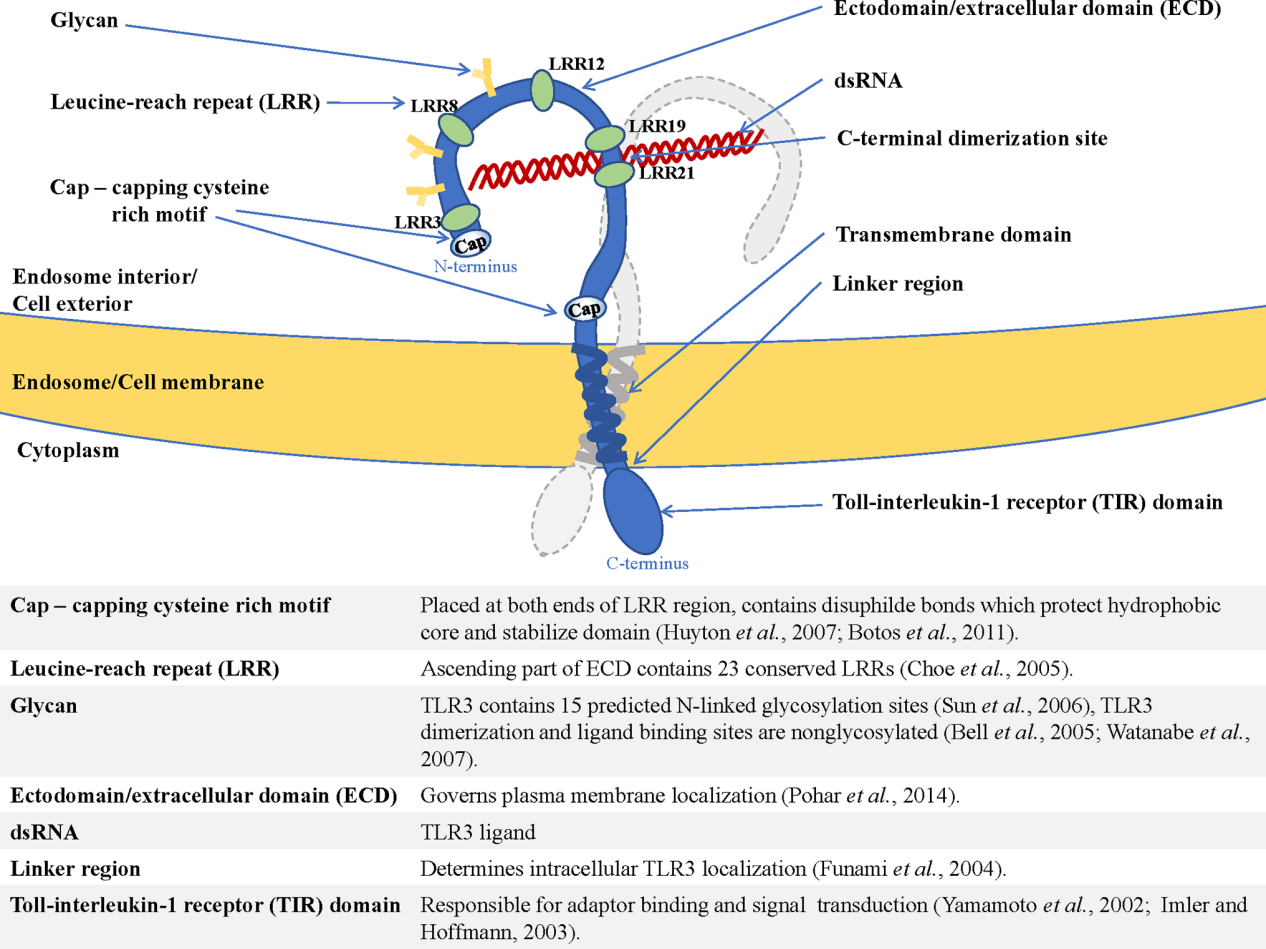
Structure of endosomal TLR localized in the endosome/cell membrane, exemplified by TLR3—shown are dimerization site, ECD (ectodomain/extracellular domain), transmembrane helix, and TIR domain (Toll-interleukin-1-receptor domain), as well as functions of the essential elements of the receptor (19–28).

The type I transmembrane proteins family comprises endosomal TLRs that are characterized by a similar structure. TLR3, TLR7, TLR8, and TLR9 contain N-terminal ectodomain (ECD) with leucine-rich repeats (LRR) involved in TLR-ligand interaction (17), and a cytosolic Toll-interleukin-1 (TIR) domain responsible for enrollment of the signaling pathway components (18). The structure of endosomal TLRs is shown in **Figure 1** and exemplified by TLR3.

Another distinguishing feature of endosomal TLRs is their presence as pre-formed dimers, e.g., human TLR9 are reported to occur in such a manner (29). Following stimulation with the TLR7 ligand, TLR7 forms an m-shaped dimer containing two ligand-binding sites (30). Interestingly, the first site is sufficient for the receptor activation, while the second site enhances the binding affinity of the ligand bound to the first site. Furthermore, each ligand binding site preferentially recognizes different moieties: guanosine or uridine-rich ssRNA, indicating that TLR7 is a dual-receptor. In the case of TLR3, dimerization is necessary for effective ligand attachment (31), and the dimerization interface is located at C-terminal 19-21 LRR components of TLR3 (LRR-CT) (32), contrary to other TLRs in which dimerization may occur in different regions of the C-terminal domain.

Following activation by the ligand, TLR7, TLR8, and TLR9 bind myeloid differentiation primary response (MyD) 88 adaptor protein through the intracellular domain, while TLR3 connects to a different adaptor protein, TIR-domain-containing adapter-inducing interferon-β (TRIF), through the TIR-domain (33). Such interactions initiate signaling cascades that promote nuclear translocation of nuclear factor kappa B (NF-κB), interferon regulatory factor 3 (IRF3), IRF7, and activator protein 1 (AP-1) transcription factors (18, 34). The ultimate goal is aimed at gene transcription and protein expression for cytokines such as tumor necrosis factor alpha (TNFα), interleukin-1 beta (IL-1β), IL-6, interferon-inducible protein 10 (IP-10), and type I interferons (IFNs) (IFN-α and IFN-β) that are able to counteract the danger raised by the invading pathogen (33).

Prototypical endosomal TLRs translocate to ligand recognition sites from ER which they populate in resting cells (35–37). Following ER residence, receptors are trafficked to the Golgi apparatus for addition of N-linked glycans, however, they may also reach the endosomes bypassing this organelle (37). TLR9 is an exception in this intracellular transportation route. After the glycosylation, the receptor is transported to the cell surface and recruits AP-2 complex to effectuate endocytosis and finally anchor in endosomes. In contrast, TLR7 recruits AP-4 complex in the cytoplasm and resettles directly from the Golgi network to endosomes (38).

Endosomal TLRs contain distinctive targeting sequences that direct the receptors to their intracellular location. Endosomal compartmentalization of TLR3 occurs due to the linker region situated between the transmembrane helix and the TIR domain (19) (**Figure 1**), while TLR7 endosomal transposition is determined by the sorting signal from the transmembrane domain (39). Interestingly, murine TLR9 is trafficked owing to the transmembrane domain (40), but human TLR9 transportation to endosomes is mediated by the tyrosine-based motif of the cytoplasmic domain (41). Folding of the adequate structure of the TLR protein may also determine its localization in the cell. For example, cysteines participating in disulfide bond formation play an important role in TLR3 stability and expression. Analysis of TLR3 mutants in the cysteines indicated that some of the modified receptors may exhibit different levels of cell surface expression (42).

## Influence of UNC93B1 on Cell Surface Localization of Endosomal TLRs

One of the accessory proteins responsible for transportation of endosomal TLRs from the ER to endosomes which ensures proper localization for effective antimicrobial immune response is UNC93B1 (20, 43). Autosomal recessive deficiency of *UNC93B1* in humans may predispose to herpes simplex encephalitis (HSE) following herpes simplex type I virus (HSV-1) infection through insufficient production of type I (IFN-α and IFN-β) and type III (IFN-γ) interferons (44). In resting cells, UNC93B1 resides in the ER (45), and upon endosomal TLR stimulation interacts with transmembrane segments of the receptors and delivers them to the ligand recognition site (46). Nucleic acids-sensing TLRs such as TLR3, TLR7, and TLR9 of mice with *Unc93B1* loss-of-function mutation are unable to leave the ER (43, 45). Furthermore, these mice are prone to infections with various intracellular pathogens (46). UNC93B1 may stabilize TLR3, TLR7, and TLR9, regulate their maturation at early state and therefore probably is responsible for the correct spatial conformation of these receptors (47). Pelka et al. propose that nucleic acid (NA)-sensing TLRs are most likely misfolded and targeted to the ER-degradation pathway in *Unc93b1^-/-^* and *Unc93b1^3d/3d^* mice due to the lack of interaction with the missing/unfunctional chaperone protein. UNC93B1 contributes to the protective role of TLR3 and TLR9 by increasing their half-life, probably through lowering their proteolytic degradation rate (48, 49). However, UNC93B1 upregulation may also increase the responsiveness of TLR3, TLR7, TLR8, and TLR9 to their agonists, and conditions that lead to increased UNC93B1 expression may yield autoimmune disorders (49).

Different proteins from the adaptor protein (AP) family have been proposed to participate in UNC93B1-mediated transition of individual TLRs to the endosomes. During TLR7 transportation aimed at ligand detection, the receptor is accompanied by UNC93B1 and AP-4, however, direct interaction has been demonstrated between AP-4 and TLR7 but not AP-4 and UNC93B1 (38). UNC93B1 also regulates TLR9 intracellular trafficking by recruiting AP-2 *via* the C-terminal domain (38), which supports clathrin-dependent internalization of TLR9 from the cell membrane (50). Knockdown of AP-2 and the exclusion of the AP-2-dependent sorting pathway of TLR9 increased TLR9 responses and TLR8 activity in HEK cells, indicating the multifaceted role of this adaptor protein (51). Interestingly, TLR3 and TLR9 are subject to regulated release from Unc93b1 in endosomes prior to ligand binding and signaling, contrary to TLR7 which binds ligand and signals while associated with Unc93b1 (52). Furthermore, following Unc93b1 phosphorylation, the Unc93b1-TLR7 complex is able to recruit Syntenin-1 for signaling termination and limiting the receptor reactivity (53).

Kanno et al. (14) observed that the appearance of TLR7 on the surface of splenic DCs occurred in a UNC93B1-dependent manner. The contribution of UNC93B1 in the transportation of TLR9 to the cell surface was reported by Onji et al. (54). Deficiency in *UNC93B1* reduced the abundance of TLR3 on the surface of the human embryonic kidney (HEK293T) cells (55), and no TLR3 was observed on the surface of murine bone marrow myeloid cells (BM-MCs) with the *Unc93B1^3d/3d^* loss-of-function mutation (56). These results indicate that UNC93B1 may be responsible for the presence or abundance of TLR3 and other endosomal TLRs on the surface of cells.

Stimulation of human umbilical vein endothelial (HUVEC) cell line with TLR3 ligand but not with TLR9 ligand, not only up-regulated *UNC93B1* mRNA expression, but also promoted TLR3 transposition to the cell membrane. Additionally, increased expression of UNC93B1 affected the transportation of TLR3 but not TLR7, TLR8, or TLR9 to the cell membrane in HEK293T cells transfected with *TLR3* and *UNC93B1*. Overexpression of UNC93B1 led to a 13-fold increase in cell surface expression of TLR3, compared to cells with endogenous expression of UNC93B1 (49). The up-regulation of UNC93B1 which increases TLR3 expression on the cell membrane could also imply an increase in intracellular/endosomal TLR3 abundance [see **Figure 4A** in (49)], however, confirmatory studies would be necessary to acknowledge such a phenomenon in cells other than HEK293T and additionally verify whether UNC93B1 can occur together with TLR3 on the cell surface. Such an interaction was revealed for uncleaved TLR9 and UNC93B1 (38). Studies on the role of UNC93B1 in the cell surface localization of TLR5 also show that although UNC93B1 mainly localizes intracellularly, it may be present in the cell membrane [see **Figure 3B** in (57)]. Whether TLR3 requires internalization from the cell surface to endosomes for triggering the signaling pathway is another issue worth investigating. Bioinformatic analyses revealed that the UNC93B1 promoter region may be regulated by poly(I:C)-induced (polyinosinic:polycytidylic acid, synthetic dsRNA) transcription factors such as IRF3, NF-κB or AP-1. Priming of cells with the TLR3 ligand may enhance responses to agonists of other nucleic acids-sensing TLRs through the up-regulation of UNC93B1 (49). These findings shed light on the dependency of TLR3 on UNC93B1 for its surface localization in cells.

N-linked glycosylation is a significant process that arranges localization and assembly and therefore determines proper endosomal TLRs signaling (58). Besides, it may be involved in TLR3 stability (59), since mutations in 2 (N247, N413) of the 15 glycosylation sites gave rise to a non-functional TLR3 (21). The addition of complex glycans to TLR3 takes place primarily in the Golgi apparatus (48), and although TLR3 is one of the most heavily glycosylated TLRs, its lateral face does not contain glycans in order to interact with dsRNA or proteins (60). The endogenous expression or simultaneous overexpression of UNC93B1 and TLR3 generates a differentially glycosylated form of TLR3 on the surface of human cell lines (48, 49), whereas such form of TLR3 was not expressed on the surface of cells with overexpressed murine Unc93b1 (20). Likely, the disparately glycosylated TLR3 may be exclusively destined for the cell membrane, but this requires further examination. Nevertheless, this feature highlights TLR3’s uniqueness, since no modified glycosylation pattern has been detected for other endosomal TLRs during UNC93B1 overexpression (49). In the work of Pohar et al. (49), it was considered that such a conservatism constitutes an evolutionary adaptation intended to protect against autoimmune response to self-nucleic acids. Interestingly, another ER resident, the protein associated with TLR4 (PRAT4A), is required for intracellular trafficking of Tlr7 and Tlr9, whose responses were abolished in PRAT4A^−/−^ BM-DCs, BM-macrophages, and splenic B cells. In contrast, Tlr3 responses were not impaired in cells from mice lacking PRAT4A (61).

Taken together, UNC93B1 is a versatile chaperone protein and not only takes part in the escape and transportation of the NA-sensing TLRs from the ER or cell membrane to endosomes, but also remains associated with TLRs for activation or termination of their signaling, and finally contributes to the generation of particular TLR forms on the cell surface. However, little is known about the delivery of cleaved forms of endosomal TLRs to the membranes of particular cell types (56), and whether this may take place in a UNC93B1-dependent manner. Cleavage of endosomal TLRs occurs in endosomes with the participation of cathepsins, important enzymes that may shape the formation of TLR-mediated immune response against pathogens (62–66). More than a dozen cathepsins have been discovered in humans, which belong to aspartic (D, E), serine (A, G), and cysteine (B, C, F, H, K, L, O, S, V, Z/X, W) proteases (67).

## Cleavage of Endosomal TLRs by Cathepsins

Compartmentalization of TLR3, TLR7, TLR8, and TLR9 is aimed at the delivery of receptors to the ligand location site. However, endosomes not only provide recognition of bacterial or viral nucleic acids but they also prevent TLRs from sensing host nucleic acids and retain the environment necessary for the activity of cathepsins, which play an important role in receptor performance (54, 64, 66, 68) (**Table 1**). Acidic pH is vital for adequate maturation of endosomes and augments ligand recognition by TLR3 and TLR9 (40, 88), whereas inhibition of acidification likely impedes the immune response (89, 90). Although pH 5.7–6.5 is optimal for TLR3 aggregation and signaling, only pH 7.5 or higher prevented the response of TLR3 to poly(I:C) in human U937 lymphocyte cell line (11). TLR3, TLR7, TLR8, and TLR9 contain individual cleavage sites and are split by various proteases (**Table 1**) into the N-terminal fragment containing part of the ECD and C-terminal fragment consisting of truncated ECD, transmembrane, and TIR domain (65, 77). Noteworthy is the fact that different cell types may have diverse proteolytic specificity and capacity, e.g., in dendritic cells (DCs), apart from “classic” TLR9 processing dependent on the cysteine protease cathepsin B and required for proper signaling, the receptor was subjected to other proteolytic events orchestrated by other enzymes (91). Cathepsin S is active regardless of an acidic environment and may cleave TLR9 between amino acids 441–470 into the 80 kDa form of an active receptor capable of inducing a signaling cascade. Processing of TLR9 between amino acids 724–735 in endosomes leads to the emergence of a soluble form. Such a soluble TLR9 (sTLR9) variant, analogous to sTLR2 and sTLR4, which occur naturally in body fluids and cellular secretions (92–94), inhibits TLR9-dependent signaling, indicating that distinctive proteolytic processes may affect TLR9 responses, an aspect that requires further investigations.

**Table 1:** Comparative presentation of endosomal TLRs and their ligands, enzymes required for cleavage, the importance of cleavage and eventual participation of cleaved fragments in signaling, and the possibility of occurrence on the surface of cells.

Endosomal TLR	Ligand	Enzymes responsible for cleavage	Cleavage required for TLR signaling	The ability of full-length/cleaved fragments to bind ligand	The requirement of C and N association for signal transduction	Occurrence on the surface of the cell
**TLR3**	double-stranded RNA	cathepsins B, L, and/or S (48), H (66)	not required (but may modulate the level of antiviral response) (48)/ required (66)	present: FL, N, C (48)	required (56, 69)	yes: CD8^+^ DCs, MZ B cells, J774 macrophages, BM-MCs (56), HEK293T cells (48), prostate epithelium (70), human conjunctival fibroblasts (HCF) (71), apical and basal plasma membrane of human endocervical cells (72), rat peritoneal mast cells (73, 74), and other
**TLR7**	single-stranded RNA, guanosine	cathepsin B* (65), asparagine endopeptidase (AEP) (75), furin-like proprotein convertases (76)	not required (64)/ required (30, 75, 77, 78)	present: FL, C (75), C (78), N, C (cooperatively involved) (30)	not required (75)/ required (30, 78)	yes: BM-DM, BM-conventional DCs, BM-plasmacytoid DCs, B cells, peripheral blood mononuclear cells (14), rat peritoneal mast cells (73, 74)
**TLR8**	uridine-rich or uridine/guanosine-rich single-stranded RNA	cathepsins*, furin-like proprotein convertase (79)	required** (68, 79)	present**: N, C (68, 79)	required** (68, 79)	unknown
**TLR9**	unmethylated CpG DNA	cathepsin B (63), F (63), K (62, 77), L (63, 64), S (63, 64, 77), AEP (80)	required (48, 64, 77, 80, 81)	present: FL, N, C (64, 77, 80)	not required (the cleaved 80 kDa form is considered to constitute a mature receptor) (80)/ required (54)	yes: conventional DCs and plasmacytoid DCs (pDCs) (54), rat peritoneal mast cells (73, 74), PBMCs (82), IECs (83), neutrophils (84), human hepatocellular carcinoma (HCC) cell lines (85), B cells (86), splenic monocytes and B cells, RAW 264.7 cells (87)

The features discussed are presented at the top of each column. FL, full-length; C, C-terminal; N, N-terminal. *studies were carried out indirectly, with the use of cathepsin inhibitor, which can exert broader activity; **result obtained on the basis of structural analysis studies.

Cleavage of TLR7 or TLR9 by cathepsins is required for signaling, contrary to TLR3, where proteolytic cleavage of the receptor may not determine the activation of the immune response (77). Despite the use of z-FA-FMK cathepsin inhibitor, TLR3 could still be activated in transiently transfected HEK293T, Huh7.5, and BEAS-2B cells in comparison to the control treatment (48), therefore, it is possible that proteolytic cleavage may untie novel functions of the TLR3 derivative forms. Compared to full-length TLR3, both C-terminal and N-terminal forms displayed longer half-life, which may influence the duration of signaling (48). Moreover, mutation of the TLR3 cleavage site or the addition of cathepsin inhibitor reduced the abundance of endosomal TLR3 destined for degradation in lysosomes. Noteworthy is that the cleaved TLR3 forms were more abundant in early endosomes, while the inhibition of cathepsin activity shifted TLR3 localization to recycling endosomes and lysosomes (48). Localization of TLR3 in various types of endosomes may have a significant impact on the signaling, as these dynamic organelles may carry the TLR3 ligand or constitute a site of receptor degradation.

Although the presence of both cleaved fragments may not be indispensable for ligand recognition among endosomal TLRs, C-terminal and N-terminal forms alone have been reported to sense their ligands (**Table 1**). Regarding TLR3, it is suggested that both forms of the receptor contain the ligand-binding domain, however, the ability to bind dsRNA by C-terminal fragment of TLR3 is ambiguous (66, 69). A certain theory postulates that similar to TLR9 (54), it is the association of C- and N-terminal TLR3 fragments which enables response to dsRNA. Cleaved TLR3 fragments are observed during the detection of cellular proteins only under denaturing conditions, which may corroborate the interaction of these forms in murine primary immune cells (56). In HEK293T cells, the deletion of 14 amino acids at the N-terminus of the C-terminal form of TLR3 suppressed immune response, probably due to exclusion of the cleaved fragments association (69). Elongation of the N-terminal receptor form by the same number of amino acids also reduced TLR3 responses (56). These observations were confirmed by experiments in which activation of NF-κB or IFN-β promoter occurred in cells where C- and N-terminal fragments were simultaneously expressed (56). Furthermore, the addition of an antibody stabilizing the interaction between C- and N-terminal forms of the receptor strengthened TLR3 signaling in endosomes. These findings strongly favor the association of cleaved TLR3 fragments, however, we cannot preclude that such cooperation is indirect, e.g., it may occur through the assistance of full-length TLR3 or other proteins. Interestingly, inherent in murine and human TLR7, cysteines of the N-terminal (C98 and C445) and C-terminal (C475 and C722) cleavage forms of the receptor are not only required for the TLR7 proteolytic processing. These unique amino acids also determine the disulphide bonds between TLR7 cleaved molecules and are indispensable for RNA sensing by the cleaved and bound forms of the receptor (78).

Notably, Qi et al. (55). observed that TLR3 mutations in P554S (situated in the region of cleavage and critical for dsRNA binding) and F303S, caused a reduction in TLR3 abundance on the cell surface, compared to wild type HEK293T cells. Earlier, Zhang et al. (95). linked P554S mutation in a patient suffering from HSE with loss of TLR3 function in central nervous system (CNS) cells and increased penetrance of the disease through insufficient antiviral response, as reviewed by Mielcarska et al. (96). Subsequently, F303S mutation was found in a patient with encephalopathy following influenza virus infection, which underlines the pivotal role of TLR3 in the antiviral defense of the brain (55). This highlights the importance of intact TLR3 cleavage site, the influence of the cleavage on ligand recognition and activation of the signaling pathway.

Collectively, different proteases have great importance in the processing of endosomal TLRs through production of active or inhibitory forms, which is continually required for the proper receptor functioning. Receptor proteolysis appears to be conserved across cell types (65), however, a thorough investigation of which enzymes contribute to regulating the TLRs signaling remains to be determined.

## Occurrence of Endosomal TLRs on the Cell Surface

Endosomal TLRs may be present in the cell membrane from where they may sense ligands. Ample surface expression of endosomal TLRs is observed in various cell lines and cell membrane-localized receptors are also capable of triggering an immune response. TLR7 may appear on the surface of cells and become a beneficial target for autoimmune therapy. For instance, in mice suffering from chronic progressive inflammation causing splenomegaly, thrombocytopenia, and chronic active hepatitis due to spontaneous TLR7-dependent systemic inflammation, symptoms were alleviated through the use of anti-surface TLR7 antibodies (14). Administration of an antibody against TLR7 in these mice inhibited the production of cytokines in immune cells such as B cells, macrophages, and DCs. Particularly, the exogenously added anti-TLR7 antibody completely blocked the production of IL-6, CCL5, and TNFα in BM-MCs, and greatly inhibited B-cell proliferation induced by the TLR7 ligand (14). Full-length and N-terminal TLR7 forms were found on the surface of immune cells such as bone marrow-derived macrophages (BM-DM) and macrophage cell lines as well as in BM-conventional DCs, BM-plasmacytoid DCs, B cells, and peripheral blood monocytes, but TLR7 in BM-derived cells was mainly localized in the intracellular compartment. Similarly, TLR7 appeared on the cell surface and intracellularly in connective tissue-type mast cells, however, exhibiting higher expression inside the cells (73). When surface TLR7 was complexed with antibodies, it was detected in lysosomes 24 h later (14). Interestingly, such an internalization process may not be associated with triggering the signaling pathway, but the degradation of the receptor.

Careful investigation of different distribution profiles of endosomal TLRs in cells may yield data on cell type-specific pathways that culminate in antimicrobial response induction. For example, following stimulation of brain cells with let-7b, a TLR7 ligand, the receptor localized to the endosomes in the cortical and hippocampal neurons which underwent apoptosis (97), and to the plasma membrane in the sensory neurons causing stimulation of the cation channel transient receptor potential A1 (TRPA1) (98). The discussed results indicate the localization of TLR7 in different types of neurons as a factor influencing the functional responses of neurons to the stimulation with the TLR7 ligand (13).

TLR8 has not been found on the cell surface thus far, but the receptor may crosstalk with other TLRs. Nucleic acid recognized by TLR8 may be of viral origin or constitute bacterial RNA released within phagosomal vacuoles (99). Total RNA of *Escherichia coli* elicited TLR7 and TLR8 activation in HEK293 cells (100), while stimulation of cell surface TLR2, TLR4, and TLR5 in human primary monocytes down-regulated TLR8-IRF5 signaling, reducing the impact of TLR8-mediated pathogen sensing (101, 102). Interestingly, human TLR8 inhibited activation of TLR7 and TLR9, likewise TLR8 from mice inhibited TLR7 activity (103). Cells from *Tlr8^-/-^* mice showed increased expression of Tlr7 and were hyperresponsive to various TLR7 ligands, resulting in the animals developing spontaneous autoimmunity (104). Furthermore, *Tlr7^-/-^* and *Tlr8-/- Tlr7-/-* mice did not show the phenotypes of *Tlr8-/-* animals, emphasizing the significant role for TLR8 control of the TLR7 expression level and its role in autoantibody production. The functional reverberations of these TLR-TLR dependencies have yet to be thoroughly investigated.

TLR9 may exist on the surface of splenic DCs (54), rat peritoneal mast cells (73), HEK293 cells following stimulation with the TLR9 ligand (36), human peripheral blood mononuclear cells (PBMCs) after the addition of LPS (82), or murine intestine epithelium after stimulation of cells with DNA from pathogenic *Salmonella enterica* (83). Present on the surface of human and murine neutrophils, TLR9 plays an important role in their activation, even after inhibition of endosomal acidification (84). Further, stimulation of TLR9 in human polymorphonuclear leukocytes resulting in their activation, culminated in enhanced expression of the cleaved functional receptor on the surface of cells. On the other hand, the forced occurrence of TLR9 on the cell surface through mutation in the transmembrane region led to inhibition of the receptor proteolysis and lethal inflammation in mice (105). Expression of cell membrane-localized TLR9 was remarkably increased on whole blood B cells of severely mechanically injured patients prone to sepsis compared with healthy controls (106). Also discovered in the plasma membrane of B lymphocytes, surface TLR9 was unable to bind its ligand, however, might negatively regulate endosomal TLR9 responses (86). It remains to be resolved how the non-ligand-binding receptor may signal from the cell surface to influence the intracellular equivalent. Interestingly, TLR9 was expressed on the surface of human HCC cell lines such as HepG2, HLE, Huh7, and SK-Hep1 (85). While full-length TLR9 was mainly expressed on the cell membrane, cleaved forms of TLR9 were abundant in the endosomes. Recently, Murakami et al. (87). confirmed the presence of TLR9 in the plasma membrane of splenic monocytes and B cells. During studies with immunocompetent cells, it was found that TLR9 surface expression varied according to the cell type as well as the status of their differentiation and activation. TLR9, together with TLR7, have been found in human airway epithelial cells (AECs), particularly in the terminal bars and cilia (107). Such an unusual pattern of expression and distribution may favor tissue-specific biological necessities.

Surface TLR3 was first observed on human fibroblast cell line MRC-5 (108). Binding of TLR3 to an antibody inhibited the poly(I:C)-mediated secretion of IFN-β by MRC-5 cells, demonstrating the functional role of the receptor on their surface. In HEK293T cells transfected to express TLR3, full-length, N- and C-terminal forms of the receptor were present on the cell surface. In contrast, the cell surface of BM-MCs subjected to similar experiment abounded in cleaved TLR3 forms rather than full-length receptor (48). These fragments were likely to be transported from endosomes, and motif-containing TLR3 plasma membrane localization dependent on UNC93B1 was assigned to the ECD of the receptor (108). Different monoclonal antibodies binding to TLR3 ECD inhibited the production of cytokines in human lung epithelial cells (109). Surface TLR3 expression was also observed in cell lines such as HUVEC, pigmented retinal epithelium (APRE-19), lung epithelium (A549), human dermal microvascular endothelium (HDMEC), stomach carcinoma (N87), and breast carcinoma (JIMT-1) (110). Recently, surface TLR3 was observed on CD8^+^ classical dendritic cells (cDCs), BM-MCs, J774 murine macrophages, and marginal zone (MZ) B cells (56). On the other hand, in monocyte-derived immature dendritic cells (MD-iDCs) and CD11^+^ blood DCs, apart from being on the surface, TLR3 was largely stored intracellularly and upon poly(I:C) stimulation the cells increased cytokine production and maturation (111). Stimulation of rat peritoneal mast cells with LL-37 peptide not only increase TLR9 expression on the cell surface but also contributed to the translocation of TLR3 from the plasma membrane to the cytoplasm (74). The peptide increased intracellular TLR3 abundance while TLR3 expression on the cell membrane decreased.

In the light of the findings discussed in the preceding section, it is justifiable to point that cell surface TLR localization is now an established scientific observation, especially in immunocompetent cells. It remains a matter of thorough investigation to discern whether such a pattern of expression has a beneficial effect for the host. Unlike self-derived ligands of TLR7, TLR8, and TLR9, the endogenous dsRNA in mammalian cells is limited to small amounts in the cytosol formed by complementary ssRNA fragments or microRNAs (miRNAs) (112, 113). The latter, although constantly synthesized by the cells, are unlikely to stimulate antiviral mechanisms. As a consequence, TLR3 present at the surface of cells in distinct organs may pose a lower risk of autoimmune response and function without pathological repercussions comparing to other endosomal TLRs (49).

## TLR3 Cell Surface Expression and Its Possible Meaning

Studies over the past several years have reported on the surface occurrence of TLR3, which may facilitate response against pathogens. This pattern of occurrence on the membranes of distinct cells appeared to go in pairs with viral infection. Therefore, a thorough examination of the cell types regarding possible cell type-specific TLR3 regulation is necessary. For instance, the TLR3 shift to the cell surface was observed after respiratory syncytial virus (RSV) infection of airway epithelial A549 cells (114), similar to the epithelium of human bronchi (BEAS-2B cells) after rhinovirus infection (115). During viral infections, dsRNA may be found as an intermediate product of virus replication in the extracellular milieu after the breakdown of the infected cells [(107), see **Figure 7** in (49) and **Figure 1** in (96)]. Abundant surface TLR3 was also detected in primary human corneal epithelial cells (HCECs), where the production of IFN-β and pro-inflammatory cytokines such as IL-6 and IL-8 was initiated after the addition of poly(I:C) (116), which also led to upregulation of surface TLR3 expression. A significant transfer of TLR3 to the surface of alveolar macrophages was observed in mice after lung contusion (LC), in comparison to the uninjured control (117). Suresh et al. demonstrated that such a process was intended to expose TLR3 to extracellular dsRNA released from injured cells. Importantly, the dsRNA-triggered downstream signaling was independent of NF-κB and type I IFNs, and led to increased macrophage apoptosis and exaggeration of the local inflammatory response which aggravated the degree of lung injury. In such a case, the discovery that the exogenous TLR3 ligand is able to mobilize membrane translocation of the receptor may indicate that ligand-induced cell priming could increase vulnerability to subsequent dsRNA recognition (118).

The issue of TLR3 function, especially, on the airway epithelial cell surface, has attracted scientific curiosity. Poly(I:C) proved to be the most effective epithelial activator in BEAS-2B and primary bronchial epithelial cells stimulated with various TLR ligands (119). Among other genes, it significantly increased the expression of *IL-8*, granulocyte-monocyte colony-stimulating factor (*GM-CSF*), and macrophage inflammatory protein-3α (*MIP-3α*), whose products foster migration and maturation of iDCs. The presence of TLR3 was also confirmed in the apical cell membrane of human tracheal epithelial cells and human AECs (107). These observations confirm the potential significance of TLR3 in defense against inhaled pathogens.

The presence of TLR3 in the cell membrane was also demonstrated in unstimulated BEAS-2B cells (109). The addition of monoclonal antibodies recognizing cell membrane-TLR3 inhibited secretion of cytokines such as IL-6, IL-8, IP-10, MCP-1, RANTES, by up to 60% after stimulation with poly(I:C). Although a significant value, it indicates that other ligand-binding sites existed on the surface TLR3 that were not blocked by the antibody, or induction of the immune response may have occurred due to activation of the endosomal receptor. Contemporaneous signaling through the surface and endosomal TLR would initiate a faster and/or more robust biological outcome, however, this remains to be further explored. Inflammation is crucial for the elimination of infections, however, excessive inflammation may be particularly harmful to the protective functions of the surface of the mucous membranes (72). Therefore, TLR3 has an essential role at the surface of epithelial cells, which constitute essential physical barriers and strengthens the notion that cell surface TLR3 is a propitious target for the regulation of TLR3 responses (56).

In lymphatic endothelial cells (LECs) such as primary human dermal (HD) LECs and lung LECs or transfected h*tert*-HDLECs, TLR3 and TLR9 occurred both intracellularly and on the cell surface (120). Interestingly, all three cell types increased the expression of ICAM-1 and VCAM-1 leukocyte adhesion molecules as well as inflammatory cytokines production in response to TLR3, but not TLR9 ligand. Similarly, primary lung LECs also increased the expression of VCAM-1 following treatment with the TLR3 ligand (120). Furthermore, poly(I:C) up-regulated the expression of ICAM-1 in HT-29 intestinal epithelial cells (IECs) (121). The stimulation effect was diminished when HT-29 cells were treated with an anti-TLR3 antibody, indicating TLR3 functionality on the cell surface. Thus, cell membrane-localized TLR3 may serve as a mediator to promote the trafficking of immune cells through the lymphatic vessels during viral incursion, which reveals a new aspect of the receptor biology.

Healthy epithelial cells of the ileum and colon serve as a defensive line of the intestinal mucosa and also express cell surface TLR3 (122). There was no difference in surface TLR3 expression between non-inflamed mucosa cells and mucosa cells from ulcerative colitis patients. However, a significant reduction of surface TLR3 in mucosa cells was found among patients with Crohn’s disease, indicating that such receptor deficiency in the intestinal epithelium may be the disease-related feature.

**Figure 2:**
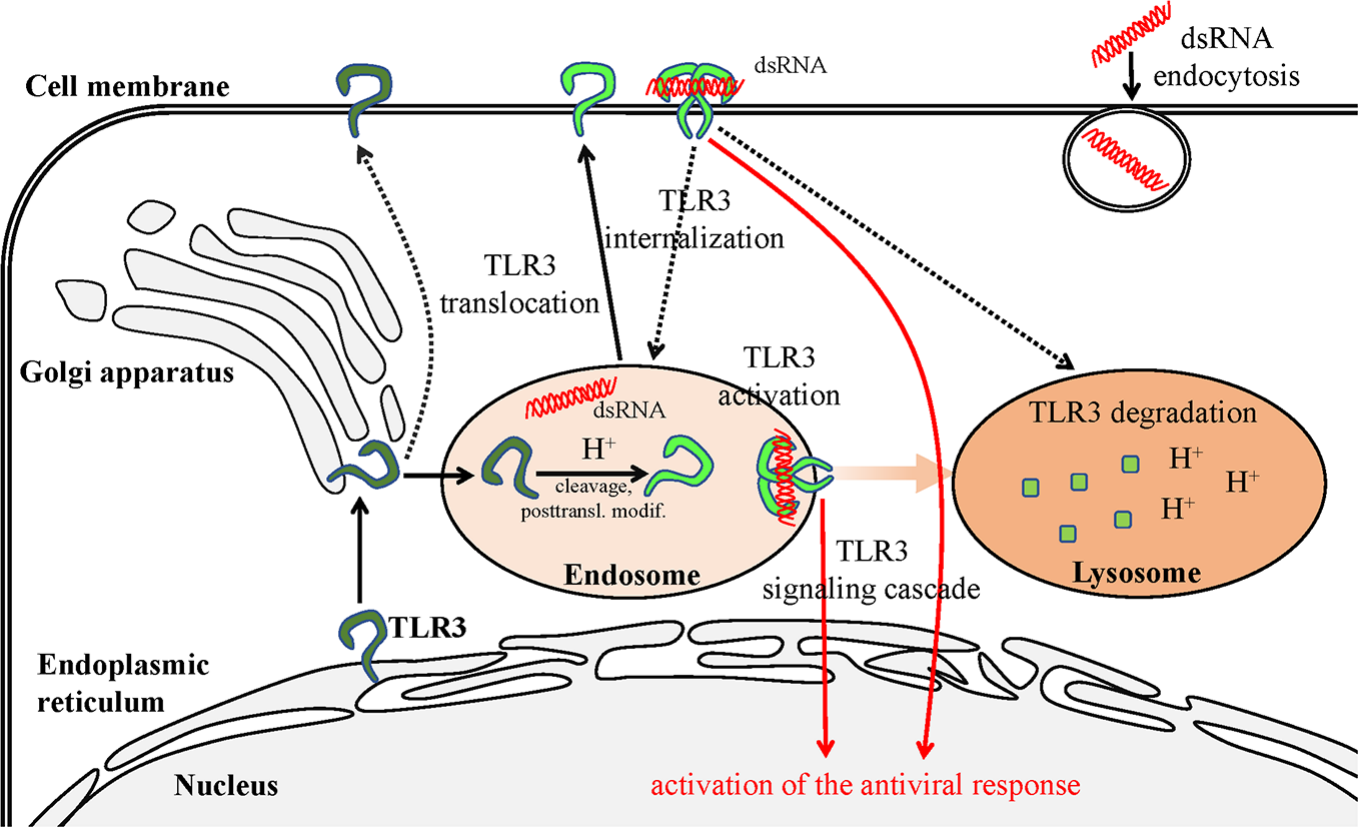
Scheme of TLR3 transportation in the cell. The transportation route is initiated in the ER and terminates in the lysosome—site of degradation. TLR3 present on the cell surface may recognize the dsRNA, which is a viral replication intermediate for some viruses, derived from necrotic infected cells. The dsRNA can also be endocytosed and recognized by TLR3 in the endosomes. Pathways requiring further examination are marked with dashed lines.

It still remains difficult to explain the role of the various forms of TLR3 on the surface of cells, although it is postulated that the cleaved receptor localized in such a way may constitute an aim for regulating the antiviral response. Murakami et al. (56). have made significant progress in investigating the possibility of dsRNA recognition and launching of protective immunity by surface TLR3 in their studies on J774 murine macrophages. In these cells, TLR3 present on the surface was mostly cleaved, indicating it may have been modified in endosomes prior to cell surface distribution. The N-terminal fragment occurred on the cell surface as the main TLR3 representative and was able to modulate the antiviral response from this particular setting. However, Murakami et al. (56). argue that surface TLR3 must be internalized in order to become activated by dsRNA. This is very likely due to the acidification which supports TLR3 activation, and because extracellularly present dsRNA, e.g., released from dead cells following viral infection, undergoes endocytosis (123). However, it cannot be precluded that the TLR3 response may be launched directly from the membrane of specific cell types by the ligand prevalent in the extracellular environment (**Figure 2**).

An example of non-beneficial localization of TLR3 on the cell surface was discovered in the metastatic derivative of IECs (15). Stimulation of IECs with poly(I:C) up-regulated UNC93B1 which also increased surface TLR3 expression. Both full-length and cleaved TLR3 forms appeared on the cell membrane, in contrast to non-metastatic cells. The inhibition of acidification in endosomal and lysosomal compartments inhibited the production of CXCL10 following TLR3 stimulation, indicating the significant role of these organelles as well as possible functions of cleaved TLR3 forms in signaling. On the contrary, inhibition of the TLR3 ligand endocytosis only slightly affected TLR3-induced CXCL10 production, however, cells failed to induce IFN-β expression. These results imply that dsRNA does not have to be absorbed into the cells for receptor activation (**Figure 2**). Furthermore, chemokine responses following stimulation of the surface TLR3 in metastatic IECs may induce a conducive environment for tumor progression (15). Although TLR3 promoted invasiveness of IECs in the discussed work, the dsRNA stimulation may entail apoptosis and reduce cell viability in various cancer types in a TLR3-dependent manner (124, 125). Therefore, careful studies of individual cancer types regarding the effects induced by cell-surface expressed TLR3 are indispensable to determine either beneficial or detrimental outcomes.

## Concluding Remarks

Localization of nucleic acid-sensing TLRs in endosomes requires maintenance of a pH suitable for cathepsin cleavage as well as potent ligand affinity and has important implications towards triggering an effective immune response. The presence of the endosomal TLRs on the cell surface may indicate an atypical condition or point to abnormal protein segregation and transportation and may affect proper degradation of the receptor. However, endosomal TLRs may also occur on the cell surface in a physiological state, and several studies point out that the localization of TLR3 or other TLRs to the cell membrane may be exploited as a therapeutic target.

TLR3 appears to exist as a functional receptor on the cell membrane more frequently than other endosomal TLRs, probably because endogenous agonists of TLR7, TLR8, and TLR9 are more abundant than dsRNA in uninfected cells, and therefore surface localization of TLR3 poses a lower risk of autoimmunity (49). Although it is believed that TLR3 activation occurs entirely in acidic endosomes (88), perhaps it would also be beneficial for cells to maintain a certain amount of TLR3 in the cell membrane in order to identify the extracellularly present viral dsRNA in case of a viral infection (**Figure 2**) (49). The probability that TLR3 ligand recognition occurs directly on the cell surface should not be disregarded, especially if the pathogen-derived ligand may not be able to reach the endosome. In such a case, it is the presence of a receptor on the cell surface that would allow the immune response to be activated. This statement is consistent with the positive effect of the exogenous dsRNA addition on the elevation of surface TLR3 expression (116) as well as increasing of TLR3 expression on the cell membrane after viral infection (114, 115). Furthermore, it underpins the significance of the surface TLR3 in mediating immune responses to viruses and should be addressed in future studies. TLR3 is engaged in recognizing dsRNA produced during the replication cycle of many viruses (95, 126–136), which may be released after lysis of infected cells.

Additional attention should be directed towards cleaved forms of endosomal TLRs, which also occur on the surface of cells and may play a role in microbial sensing. UNC93B1 is a protein indispensable for proper signaling of endosomal TLRs, however, mechanisms by which it may modulate surface TLR transportation and be involved in trafficking of the cleaved TLR forms await further studies.

The possibility that endosomal TLRs may occur in the cell membrane and act as stable and functional receptors seems particularly interesting. Already, investigations reveal that localized in such a way, these receptors may become disease-conducive or act as salutary immune sensors. Consequently, NA-sensing TLRs present on the plasma membrane may serve as therapeutic targets for functional monoclonal antibodies and might account for the progression of new therapeutical approaches towards rare human diseases that are difficult to treat. Discovering pathways originating at the cell surface may uncover new functions of endosomal TLRs, as well as subserve in better understanding individual aspects of their activation.

## Author Contributions

All authors listed have made a substantial, direct, and intellectual contribution to the work and approved it for publication.

## Funding

This work was supported by the National Science Centre Poland [grant number UMO-2016/23/N/NZ6/02499].

## Conflict of Interest

The authors declare that the research was conducted in the absence of any commercial or financial relationships that could be construed as a potential conflict of interest.

## Abbreviations

AECs, airway epithelial cells; AEP, asparagine endopeptidase; AP-1, activator protein 1; BM-DM, bone marrow-derived macrophages; BM-MCs, bone marrow myeloid cells; cDCs, classical dendritic cells; CNS, central nervous system; DAMPs, danger-associated molecular patterns; DCs, dendritic cells; dsRNA, double.
